# Abstaining from symptomatic implants of modified tension band wiring by nonabsorbable suture fixation for transverse patella fractures

**DOI:** 10.1186/s13018-021-02494-3

**Published:** 2021-06-09

**Authors:** Puripun Jirangkul, Arkaphat Kosiyatrakul

**Affiliations:** Department of Orthopaedics, Phramongkutklao Hospital and College of Medicine, Bangkok, 10400 Thailand

**Keywords:** Transverse patella fracture, Nonabsorbable suture, FiberWire, Modified tension band wiring, Symptomatic implant

## Abstract

**Background:**

Modified tension band fixation has become commonly used for transverse patella fractures. The conventional stainless steel wire provides sufficient stability but may be associated with complications.

**Objective:**

The study aimed to evaluate the effectiveness of a new modified tension band fixation technique for transverse patella fractures using a nonabsorbable suture.

**Material and methods:**

We present the result of a prospective series using a nonabsorbable suture (FiberWire) for transverse patella fractures. The mean follow-up period totaled 12 months. A total of 16 patients were evaluated by radiographic and clinical review. The postoperative clinical evaluation employed Lysholm and Böstman scores.

**Result:**

All clinical results on follow-up were good to excellent. Minimal intra-articular joint stepping and further fracture displacement were recorded. No patient needed re-operation, and functional outcomes of the knee were satisfactory. No significant differences were found between the injured and contralateral knee range of motion. No symptomatic implants and skin complications were noted, and all fractures were completed heal within 15 weeks.

**Conclusion:**

FiberWire provided sufficient stability and reduced postoperative complications. The results proved appropriate, and the technique has merit, as it obviates the need for re-operation.

## Background

Patella fracture incidence is relatively uncommon, representing approximately 0.5 to 1.5% of all skeletal injuries [1, 2]. Two different mechanisms can cause patella fractures. The most common is a direct impact on the anterior side of the knee. The remaining cases are caused by indirect injury with eccentric contraction of the forceful quadriceps muscle. One-third of those patients require a surgical intervention [3]. Conservative treatment methods with knee splinting are recommended for nondisplaced or minimally displaced fractures with intact extensor mechanisms [4]. Surgical treatments are considered when the fracture displacement exceeds 3 mm or the articular incongruity exceeds 2 mm [3].

Historically, a modified tension band wiring technique was developed in the 1950s by the Arbeitsgemeinschaft für Osteosynthesefragen/Association for the Study of Internal Fixation (AO/ASIF). This technique for transverse fractures is ideal because it uses the braces’ compression during bending movements, allowing early mobilization. A tension band converts tension from the muscle pull to compression at the articular side of the fracture. This stage enables improved fracture union, as absolute stability is provided when interfragmentary compression is induced on a bending fracture [5].

Although the common use and sufficient stability of standard tension band wiring, symptomatic implants, and skin complications such as infection have been reported in the literature, removal of troublesome material is needed in up to 60% of these patients [3, 6, 7]. An alternative material to replace the metal wire is nonabsorbable suture (FiberWire/Ethibond), producing similar outcomes with a lower complication rate [8–11] and comparable strength in a biomechanical study [12]. We present a new surgical technique of modified tension band wiring of transverse patella fractures. All steps of the operation were strictly performed following the Arbeitsgemeinschaft für Osteosynthesefragen/Orthopaedic Trauma Association (AO/OTA) principle of modified tension band wiring using FiberWire, instead of Kirschner wires (K-wires) and stainless steel wire.

## Material and methods

Between January 2017 and January 2019, 25 patients who sustained simple displaced transverse patella fractures with a disrupted extensor mechanism were enrolled in our study. Surgery was performed within 14 days after injury. An experienced orthopedic trauma surgeon evaluated the fracture configurations, radiographic evidence, and clinical outcomes. Patient demographics including age, sex, body mass index (BMI), details of the injury mechanism, smoking status, and timing to surgery were recorded (Table 1). We excluded 1 patient medically unfit for surgery, 3 patients with open fractures, 3 patients associated ipsilateral limb fractures, and 2 patients with previous history of ipsilateral knee surgery. Nonoperative management was used for the patient medically unfit for surgery. The remaining eight cases were managed by tension band wiring with conventional stainless steel wires. This study was approved by the Institutional Review Board and the Medical Ethics Committee, Royal Thai Army Medical Department, Thailand. Informed consent was obtained from all patients.

**Table 1:** Baseline characteristic of 16 patients

**Age (y)**	
Mean ± SD	45.18 ± 19.14
Median (min - max)	41 (19 - 84)
**Sex – n (%)**	
Male	6 (37.50)
Female	10 (62.50)
**BMI (kg/m**^**2**^**)**	
Mean ± SD	25.50 ± 4.47
Median (min - max)	25.18 (18.42 - 33.10)
**Mechanism**	
Direct	15 (93.75)
Indirect	1 (6.25)
**Smoking**	
No	13 (81.25)
Yes	3 (18.75)
**Timing to surgery (d)**	
Mean ± SD	8.88 ± 2.96
Median (min - max)	8.50 (5 - 14)
**Flexion of the normal side (degree)**	
Mean ± SD	128.44 ± 12.87
Median (min - max)	130 (105 - 155)

SD indicates standard deviation

### Surgical technique

Materials used for fixation were the double-strand No.5 FiberWire sutures (Arthrex, Naples, FL, USA). FiberWire was characterized by a core of several small, individual strands of ultra-high-molecular-weight polyethylene covered with braided polyester suture material. Related literature using the three-point-bend test revealed double-strand FiberWire exhibited significantly higher failure load than stainless steel wire [13].

**Fig. 1:**
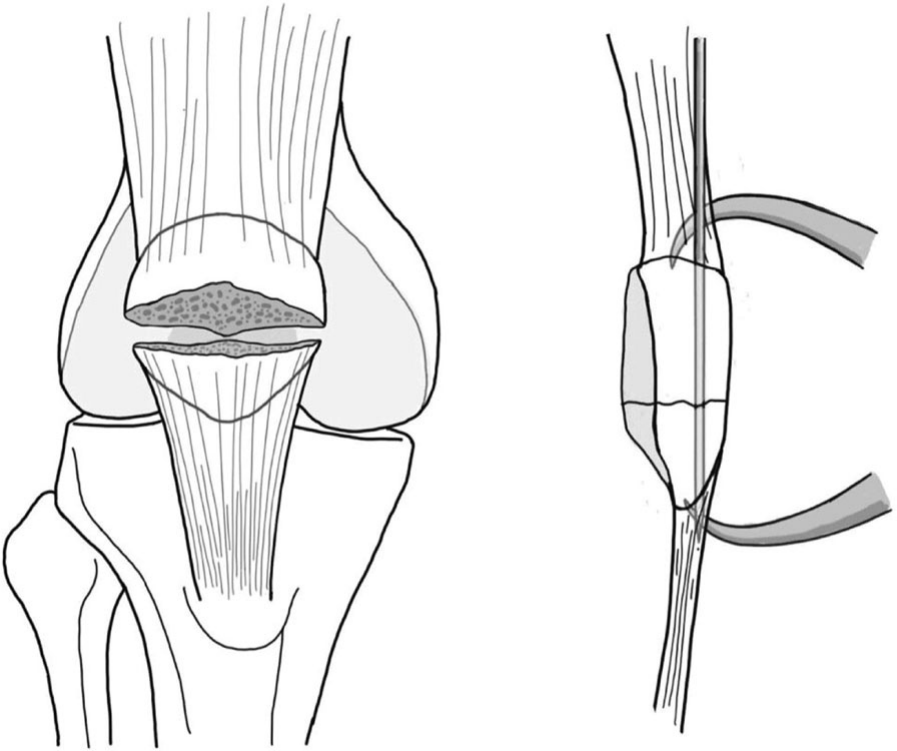
The transverse patella fracture can be reduced with patella reduction forceps and held by longitudinal K-wires

**Fig. 2:**
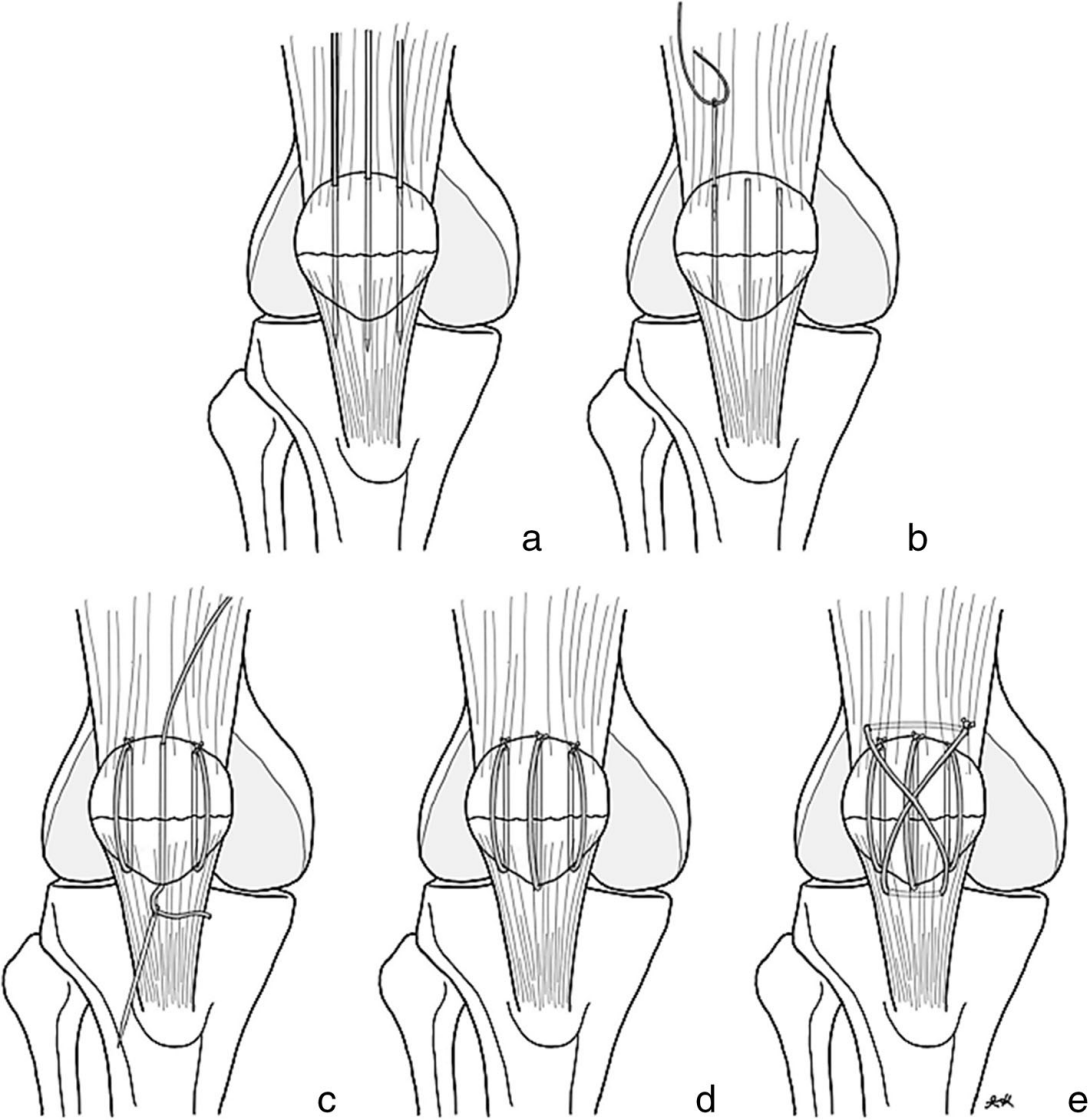
Transverse fracture is held by 3 K-wires (**a**). A FiberWire with Beath needle is passed through 3 longitudinal drill holes (**b**, **c**). Three longitudinal loops FiberWire were tied down (**c**, **d**). A figure-of-eight fashion of FiberWire is provided (**e**)

**Fig. 3:**
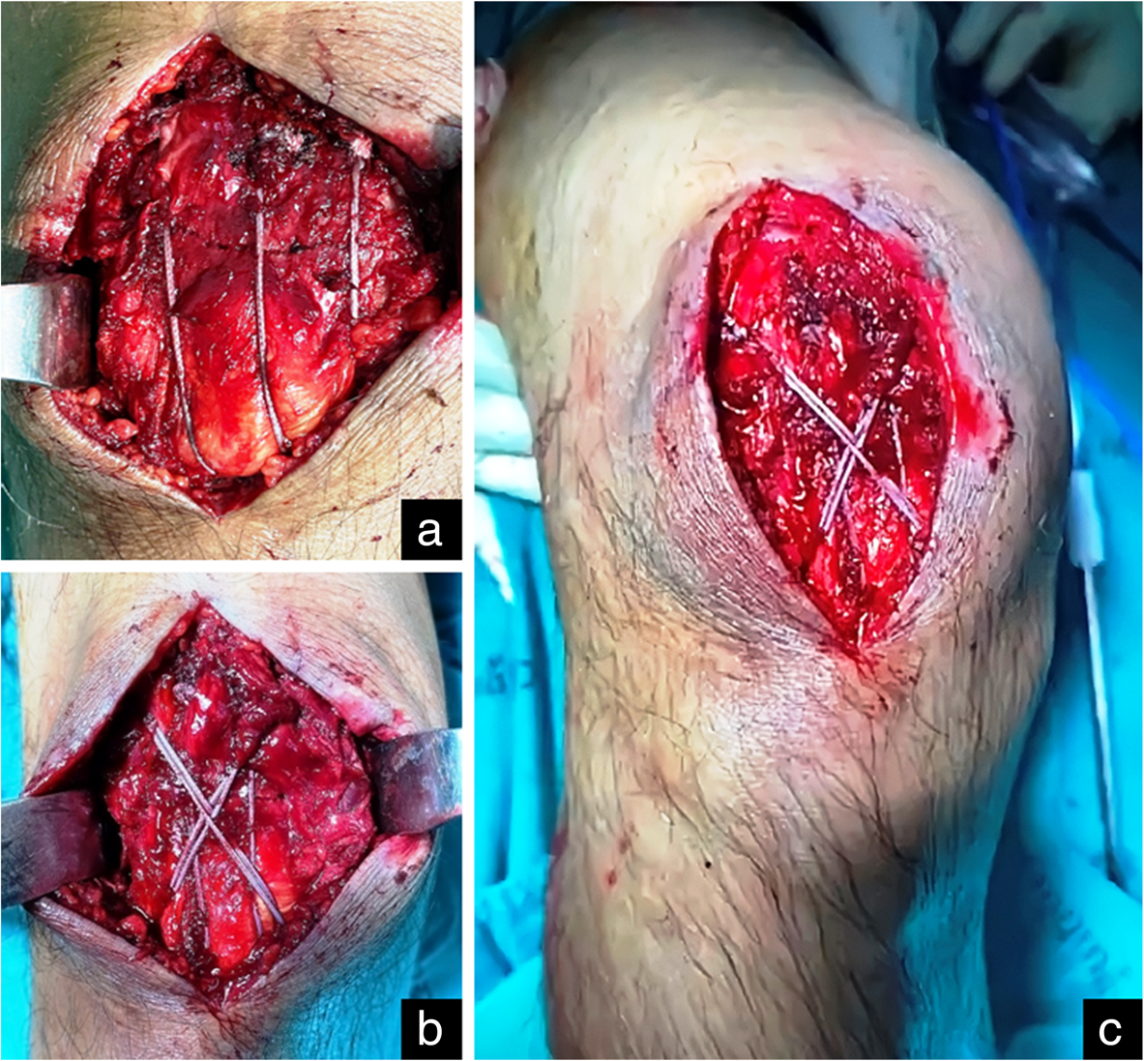
Intra-operative finding showed the transverse patella fracture underwent open reduction and fixation with the modified tension band technique using FiberWire. Three longitudinal loops were tied down (**a**). Figure-of-eight loops crossed over the patella’s outer cortex (**b**). Intra-operative stability fixation was assessed; no fracture gap on passive ROM 0–90° was detected (**c**)

Initially, all patients were managed with a leg back slab with necessary medication and prepared for surgery. Operation was performed after acute swelling had subsided. The patient was placed in the supine position, in either spinal or general anesthesia, and prophylactic intravenous antibiotic before surgery was administered. A tourniquet was placed around the upper part of the patient’s thigh and a straight longitudinal surgical incision was employed. After the fracture site was identified, fracture reduction was performed using patella reduction forceps (Fig. 1). Caution included iatrogenic fracture while applying the reduction forceps especially fixation in osteoporotic bone. A small external lateral arthrotomy was applied to allow finger palpation of the articular surface to assess the accuracy of the reduction. After the satisfactory intra-articular reduction was accomplished, a simple transverse fracture could be held accurately using three parallel 2.0-mm K-wires through the fracture line (Figs. 1 and 2a). The longitudinal drill holes should be made accurately to avoid the cutting bone effect of suture fixation. K-wires were then removed and FiberWire was passed through the three longitudinal drill holes using a Beath needle (Fig. 2b). Three longitudinal looped FiberWire strands were tied down over the patella’s outer cortex with non-sliding knots (Figs. 2c, d and 3a). To obtain an anterior tension band, a second No.5 FiberWire was inserted deep into the quadriceps tendon through the wire passer, crossed over the patella’s outer cortex in a figure-of-eight fashion, and was then passed beneath the patellar tendon (Fig. 2e). The suture could be avoided to cut the tendon by inserting FiberWire deep into the total surface area of the quadriceps and the patellar tendon, which is similar to the standard modified tension band technique. The non-sliding knot was tied over the patella’s outer cortex and buried in the soft tissue. To provide a secure fixation, we performed a double figure-of-eight fashion using FiberWire loops (Fig. 3b). After bony fixation was completed, intra-operative stability of fixation was assessed by flexing the knee to 90°. No fracture gap was detected during passive range of motion (ROM) (Fig. 3c). The joint capsule, quadriceps retinaculae, and the fascia were meticulously repaired with vicryl sutures. The skin was closed with interrupted mattress sutures and a compression bandage was applied.

### Postoperative protocol

Typically, after secure fixation by modified tension band wiring, no need exists for knee immobilization. Both active and passive knee motion can begin on the first day postoperatively. We used the hinged knee brace in our practice and locked the knee motion from 0 to 30° for 4 weeks. The patients were allowed to bear their weight as tolerant, using crutches while walking. Isometric exercises including quadriceps training were initiated to induce interfragmentary compression and regain the quadriceps’ power.

At the follow-up clinic, the knee’s anteroposterior and lateral radiographs were obtained at 2- to 4-week intervals until evidence of bone union was achieved and at 3-month intervals until final follow-up. Stitches were removed 2 weeks after surgery. Later, when the knee splint was removed, full knee motion was started using precise quadriceps exercises.

### Outcomes’ assessment

Clinical results were assessed at 3, 6, and 12 months postoperatively by 2 senior residents in orthopedics. Radiographs were evaluated by the author who performed the surgeries and radiologist using a picture achieving and communication system. Fracture healing was clinically defined as no pain or tenderness on movement and weight-bearing over the fracture zone [14]. Radiographic fracture union was defined based on haziness with consolidating the minimal three cortical sides connecting the fracture fragment [14, 15]. More than 3 mm postoperative fracture displacement or the articular incongruity that exceeds 2 mm was defined as unsatisfactory fixation. We assessed the postoperative ROM difference by comparing ROM of the injured knee with the contralateral side. The postoperative knee flexion and the ROM difference were evaluated at 3, 6, and 12 months postoperative. The functional outcomes were evaluated according to the Lysholm [16] and the Böstman scores [17], including residual pain, knee motion, quadriceps strength, and ability to return to regular activities to quantify knee function objectively. The postoperative knee motion and the functional knee scores were assessed using one-factor ANOVA followed by Bonferroni correction for multiple comparisons with statistical significance set at the .0167 level. Timing to fracture union, further displacement of fractures, complications, and the needs for re-operation were also recorded.

## Results

A total of 16 patients were recruited in this study. Patient demographics are shown in Table 1. Our patients attended the follow-up clinic for a minimum of 12 months. No patient lost their follow-up. The mean follow-up period was 14.6 months (range, 12–18).

### Clinical outcomes

**Fig. 4:**
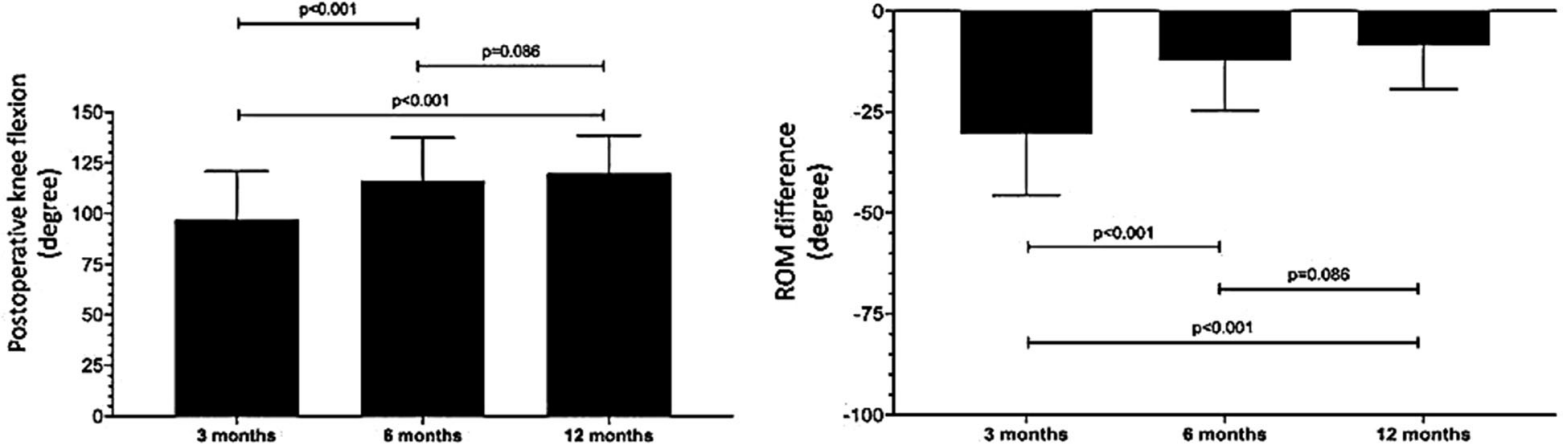
Postoperative knee flexion and ROM difference between the injured knee and the contralateral side at 3, 6, and 12 months of follow-up period. Adjustment for multiple comparisons: Bonferroni. The mean difference is significant at the .0167 level

**Fig. 5:**
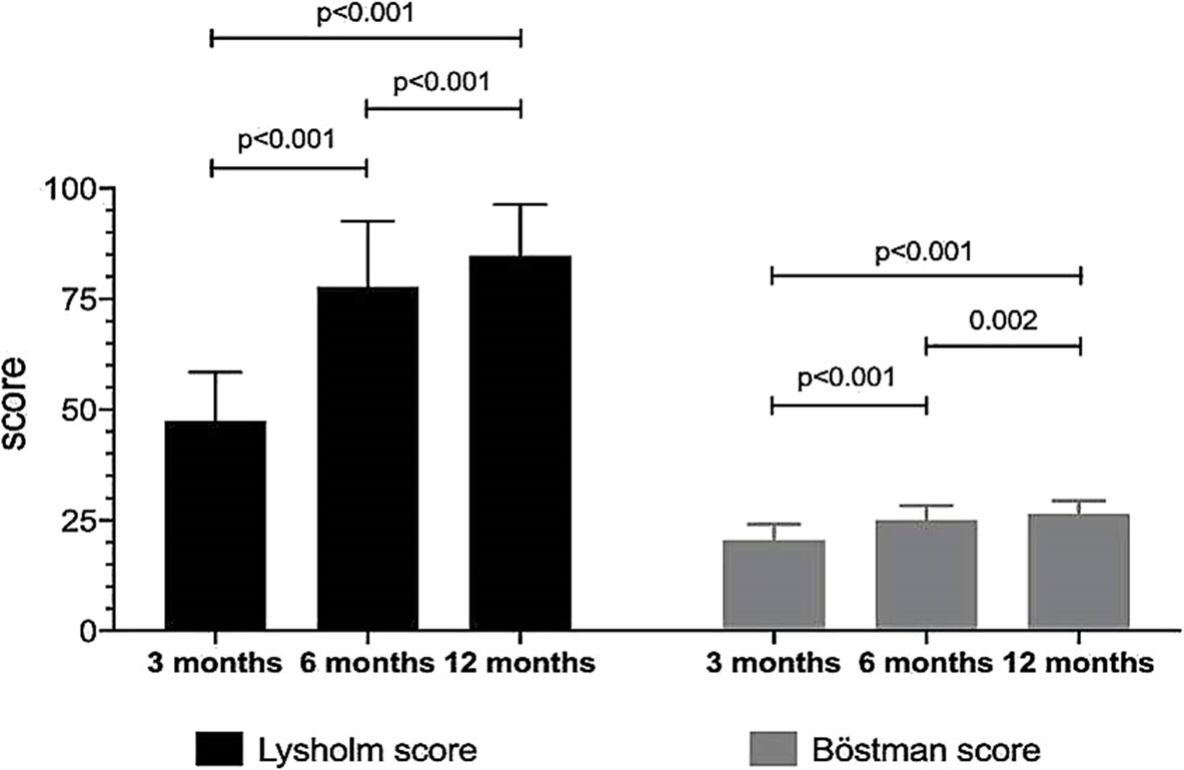
The postoperative functional knee scores at 3, 6, and 12 months of follow-up period. Adjustment for multiple comparisons: Bonferroni. The mean difference is significant at the .0167 level

The postoperative knee flexion and the ROM difference were evaluated. The statistically significant differences were observed between the knee motion at 3 months (baseline) and at 6 and 12 months postoperative (Table 2). However, no significant differences were found between 6 and 12 months postoperative knee motion (Fig. 4). The functional outcomes were objectively evaluated using Lysholm and Böstman scores at 3, 6, and 12 months of follow-up (Table 3). One-factor ANOVA with Bonferroni post hoc testing demonstrated significant differences of the postoperative functional knee scores between each follow-up period (Fig. 5). All cases achieved good to excellent outcomes (Table 4). Unsatisfactory results were defined as Böstman score less than 20 points, good was 21 to 27 points, and excellent was 28 to 30 points.

**Table 2:** Postoperative knee flexion and ROM difference between the injured knee and the contralateral side

***Postoperative knee flexion***			
**Follow-up period**	**Degree of flexion**	**Mean difference**	*p*-value*
	**(mean ± SD)**	**(95% CI)**	
3 months	96.88 ± 24.01		
6 months	116.25 ± 21.25	19.38 (13.23–25.52)	<0.001*
12 months	120.00 ± 18.62	23.13 (16.39–29.86)	<0.001*
Repeated measures analysis of variance	–	–	<0.001**
***ROM difference***			
**Follow-up period**	**ROM difference**	**Mean difference**	*p*-value*
	**(mean ± SD)**	**(95% CI)**	
3 months	−30.31 ± 15.33		
6 months	−12.19 ± 12.38	−18.13 (−23.53, −12.72)	<0.001*
12 months	−8.44 ± 10.91	−21.88 (−28.17, −15.58)	<0.001*
Repeated measures analysis of variance	–	–	<0.001**

*SD* standard deviation, *CI* confidence interval

*Paired t test comparison with baseline (3 months)

**One-way repeated measures ANOVA

**Table 3:** Postoperative functional knee scores of 16 patients at 3, 6, and 12 months

***Lysholm score***			
**Follow-up period**	**Lysholm**	**Mean difference**	*p*-value*
	**(mean ± SD)**	**(95% CI)**	
3 months	47.44 ± 11.02		
6 months	77.75 ± 14.83	30.31 (25.80–34.82)	<0.001*
12 months	84.75 ± 11.62	37.31 (33.96–40.66)	<0.001*
Repeated measures analysis of variance	–	–	<0.001**
***Böstman score***			
**Follow-up period**	**Böstman**	**Mean difference**	*p*-value*
	**(mean ± SD)**	**(95% CI)**	
3 months	20.56 ± 3.58		
6 months	25.00 ± 3.29	4.44 (3.30–5.57)	<0.001*
12 months	26.44 ± 3.01	5.88 (4.71–7.04)	<0.001*
Repeated measures analysis of variance	–	–	<0.001**

*SD* standard deviation, *CI* confidence interval

*Paired t test comparison with baseline (3 months)

**One-way repeated measures ANOVA

**Table 4:** Clinical outcomes of 16 patients at 12-month follow-up period by Böstman score

Böstman score	N	Percentage (95% CI)
Unsatisfactory (< 20)	0	–
Good (20 to 27)	8	50% (24.65–75.35)
Excellent (28 to 30)	8	50% (24.65–75.35)

*CI* confidence interval

### Radiographic outcomes

**Fig. 6:**
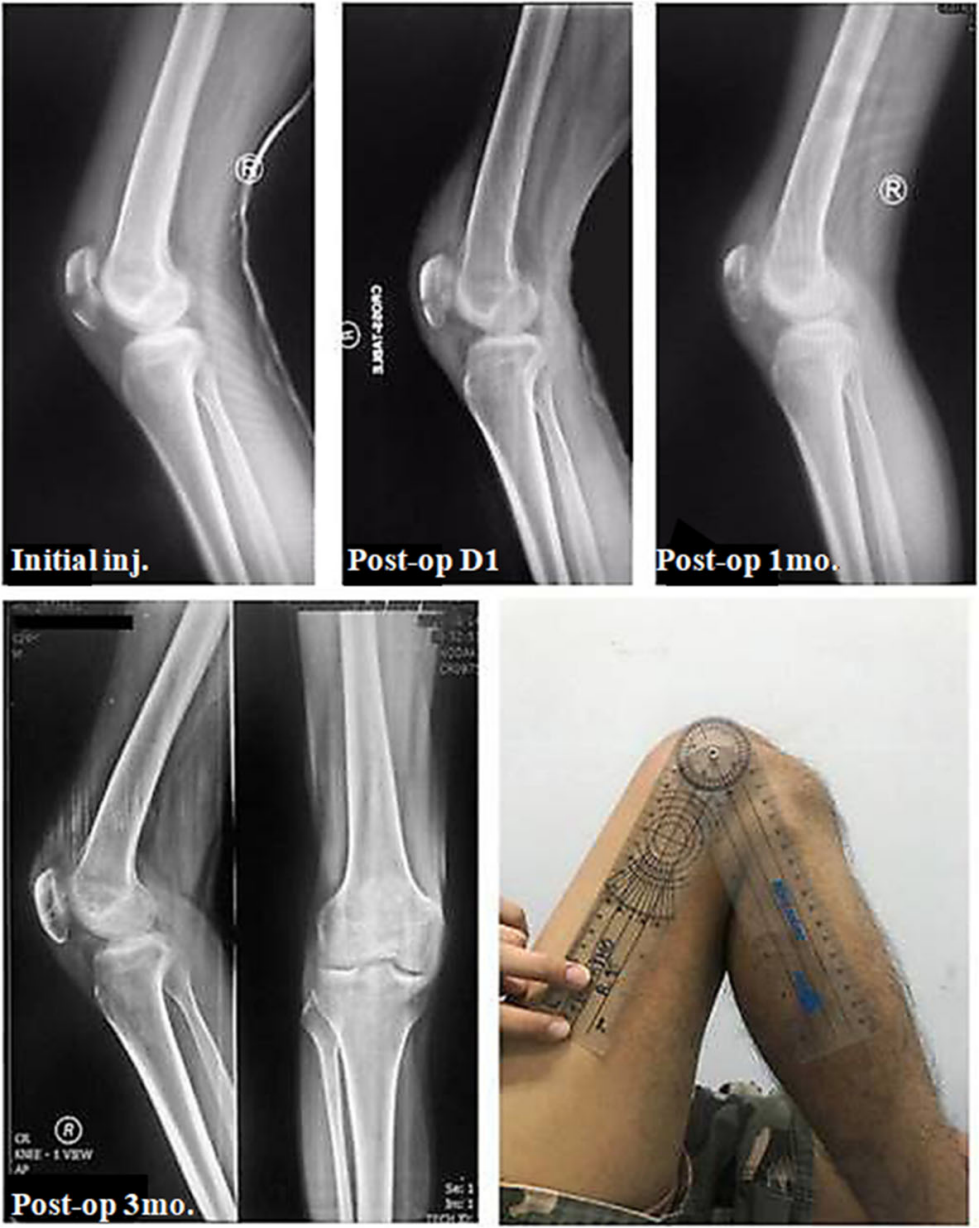
A 29-year-old male suffering from an indirect injury of the right patella. The initial radiograph shows a displaced transverse fracture of the patella. Postoperative radiographs reveal no intra-articular stepping and no displacement of the fracture site. The fracture healed within 3 months. Physical examination shows 0–130° of knee motion without pain. The ROM difference is −20°. Eventually, the patient gained full motion in 6 months

**Fig. 7:**
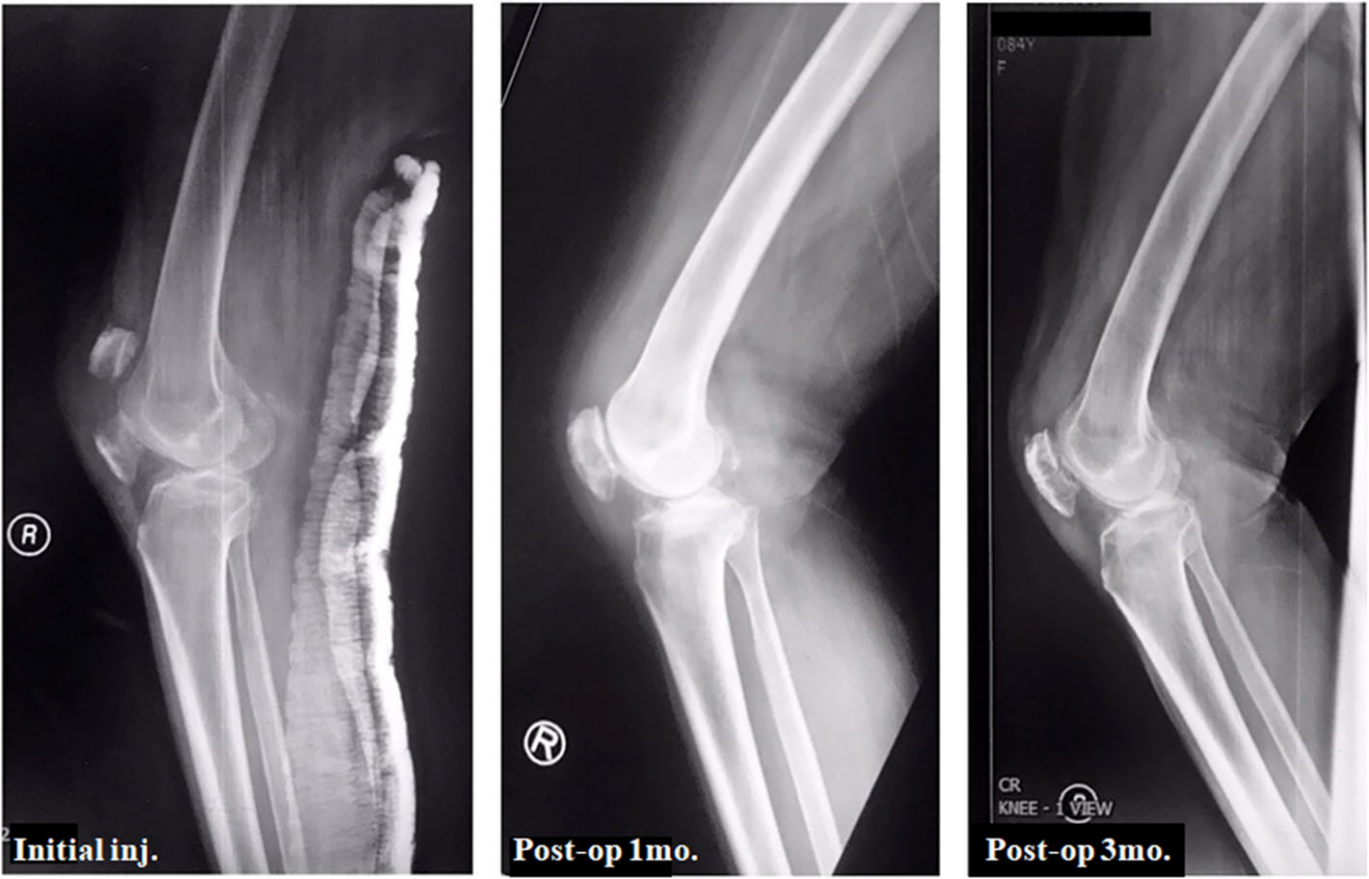
An 84-year-old female with underlying primary osteoarthritis and postmenopausal osteoporosis experiencing direct injury to the right patella. The initial radiograph reveals an articular displaced fracture of the right patella. A bone union is seen in 3 months postoperative radiograph without complications

All fractures achieved a complete fracture union (Figs. 6 and 7). The bone union time averaged 11.68 weeks (range, 8 to 15). No progressive traumatic osteoarthritis of the injured knee was found in our series.

### Complications

No medical complications were recorded during surgery and in the early postoperative period. No symptomatic hardware, skin complications, delayed union, and nonunion were found among our patients. No patient needed hardware removal or re-operation. No iatrogenic comminuted patella fracture was noted. Only one patient, a 28-year-old male, had further fracture displacement on the patella’s outer cortex. This patient took off his knee splint when discharged and initiated aggressive knee motion, such as squatting exercise and deep knee flexion. The outer cortex, namely the tension side, displaced 1.5 mm at a 2-week follow-up while the compression side remained intact. Re-operation was unnecessary because no intra-articular stepping was involved. We suggested him not to remove the knee splint and stop aggressive knee exercise. Eventually, fracture union was achieved at 14 weeks without other complications.

## Discussion

The objectives of operative treatment are reducing the anatomic articular surface and restoring the knee extensor mechanism. Internal fixation maintains fracture reduction until the fracture has healed. Even though standard stainless steel wire can provide stable fixation, all techniques using metal wires are associated with symptomatic hardware and other complications [18]. Thus, a high rate of re-operation or hardware removal is reported.

Currently, many alternative materials are available for transverse patella fracture. This study selected FiberWire. Although the strength of FiberWire is weaker than that of stainless steel wire, the maximum tensile force is more potent [13]. Related studies using FiberWire in transverse patella fracture fixation show a low symptomatic hardware removal [9–11]. Busel et al. reported 8% symptomatic hardware removal after patella fixation using cannulated lag screws and FiberWire [10]. Camarda et al. reported a retrospective case series using only No.5 FiberWire tension band. In their study, the authors performed a peripatellar circumferential cerclage close to the bone followed by a figure-of-eight FiberWire loop [9]. Although no metal implant was retained, its principle was not the actual modified tension band fixation due to the lack of parallel longitudinal intra-osseous fixation. Other related studies reported using the modified tension band technique with FiberWire and retained metallic parallel implants including K-wires [19] and cannulated lag screws [10]. Although a low rate of symptomatic hardware removal was reported, the retained metal hardware may irritate soft tissue or implant migrations and encounter breakage. Li et al. advocated double fixation using bio-absorbable cannulated lag screws and braided polyester suture tension bands [20]. This surgical technique provides a rigid stable fixation without metal hardware retention. The authors used bio-absorbable lag screws instead of parallel K-wires.

Our technique provided stable fixation of the patella fracture without any retained metal hardware. According to the standard modified tension band wiring technique which is performed by drilling two parallel K-wires and placing a figure-of-eight loop of stainless steel wire anteriorly [21, 22]. We followed the principle of standard modified tension band wiring using FiberWire, instead of metal wire. In our opinion, intra-osseous longitudinal fixation by screws may cause iatrogenic comminuted patella fractures especially among the elderly with underlying severe osteoporosis. In our study, three longitudinal intra-osseous FiberWires were applied instead of two parallel K-wires (Fig. 2d) and following applied FiberWire in a figure-of-eight fashion (Fig. 2e). Actually, only the single figure-of-eight FiberWire loop may have sufficient tensile force resistance, but we obtained more rigidity by adding another figure-of-eight loop (Fig. 3).

We suggest applying the postoperative hinged knee brace with limit deep knee flexion for 2 to 4 weeks. The patients should avoid early aggressive knee exercises. If aggressive knee exercise and deep knee flexion are initiated while the bone healing process is still incomplete, it may be technically possible to break the FiberWire or loosen the surgical knots. This point, in turn, elevates the risk of fixation failure and further loss of fracture reduction.

Our study possessed some limitations. First, our technique was not compared with the standard modified tension band wiring technique, so a further comparison study may be required. Second, our population size was modest compared with extensive national studies. Third, the follow-up period was relatively short. Long-term results and complications, including traumatic osteoarthritis of the knee, should be observed. Fourth, this study used plain radiographs to assess fracture union. However, it would have been better to evaluate the accurate timing to fracture union using computer tomography. Further comparison studies should be advocated in terms of clinical outcomes, risks, and benefits; postoperative complications and re-operation rates would include those occurring during the learning curve. Large sample size and lengthy follow-up may delineate more conclusive opinions.

## Conclusion

In conclusion, FiberWire is a nonabsorbable suture that is familiar and convenient to use among orthopedic surgeons. The modified tension band fixation of the patella using FiberWire not only could be easily performed but is also reproducible. However, this suture fixation method is limited to simple displaced transverse patella fractures. Importantly, no metal implant retention was found using this surgical technique, which would reduce complications, including skin irritation, skin infection, hardware migration, and breakage. Symptomatic hardware removal is unnecessary.

## Data Availability

All the data are available in contact with the corresponding author.

## Abbreviations

AO/ASIF: Arbeitsgemeinschaft für Osteosynthesefragen/Association for the Study of Internal Fixation
AO/OTA: Arbeitsgemeinschaft für Osteosynthesefragen/Orthopaedic Trauma Association
BMI: Body mass index
CI: Confidence interval
K-wires: Kirschner wires
ROM: Range of motion
SD: Standard deviation
